# Long‐term quality of life after liver transplantation for non‐resectable colorectal metastases confined to the liver

**DOI:** 10.1002/bjs5.50116

**Published:** 2018-10-25

**Authors:** S. Dueland, P.‐D. Line, M. Hagness, A. Foss, M. H. Andersen

**Affiliations:** ^1^ Department of Oncology, Oslo University Hospital Oslo Norway; ^2^ Department of Transplantation Medicine, Oslo University Hospital Oslo Norway; ^3^ Institute of Clinical Medicine, University of Oslo Oslo Norway; ^4^ Institute of Health and Society, University of Oslo Oslo Norway

## Abstract

**Background:**

Liver transplantation for patients with non‐resectable colorectal liver metastases offers increased survival, with median overall survival of more than 5 years. The aim of this study was to compare quality of life before and up to 3 years after liver transplantation for colorectal liver metastases.

**Methods:**

Quality of life was assessed using the European Organisation for Research and Treatment of Cancer QLQ‐C30 questionnaire version 3.0. The patients received the questionnaire before and up to 3 years after liver transplantation.

**Results:**

Some 23 patients were included in the analysis. Three months after liver transplantation they reported reduced quality of life (global health status scale), physical function and role function, and increased dyspnoea. At 6 months, global health status, physical function and role function had returned to pretransplant values. Three years after liver transplantation all symptom and function scores were comparable to baseline values. Patients with high scores for fatigue, pain and appetite loss at baseline had reduced 3‐year overall survival.

**Conclusion:**

Patients with non‐resectable colorectal liver‐only metastases receiving liver transplantation had good long‐term quality of life. Patients with high symptom scores before transplantation had reduced 3‐year overall survival.

## Introduction

Colorectal cancer is one of the most common malignancies in Western countries and a leading cause of cancer‐related death^1^. Many of these patients present with or develop metastases, most commonly affecting the liver^2^. Hepatic resection is considered the only curative treatment, with a reported 5‐year overall survival (OS) rate after resection of about 40 per cent. Only about 20 per cent of patients with colorectal liver metastases (CRLM), however, are candidates for liver surgery and the majority will develop further recurrences^3^. The standard treatment option for most patients with metastatic disease from colorectal cancer is palliative chemotherapy, with median OS of about 2 years from the start of chemotherapy and a 5‐year OS rate of about 10 per cent^4^. Progression‐free survival after the start of first‐line chemotherapy is less than 12 months^5^. Patients with liver metastases that become resectable after chemotherapy have increased survival compared with those who have non‐resectable disease^6^. Improved response rates to chemotherapy regimens have been associated with increased resection rates, and better progression‐free survival and OS. This may be further enhanced by the use of antiepidermal growth factor receptor antibodies^7^ ^8^.

Liver transplantation (LT) is the standard treatment in patients with end‐stage liver failure, and is offered widely to selected patients with primary liver cancers and liver metastasis from neuroendocrine tumours^9^, ^10^, ^11^, ^12^, ^13^, ^14^. LT for malignant tumours accounts for about 16 per cent of all LTs in the European Liver Transplant Registry^15^. The shortage of donor livers led to the abandonment of LT for CRLM owing to poor survival^16^ ^17^. The present authors^18^ have previously reported a 5‐year OS rate of 56 per cent in patients with non‐resectable colorectal metastases confined to the liver receiving LT, compared with 9 per cent in patients treated with chemotherapy. LT is a major surgical procedure and major postoperative complications have been described after transplantation in patients with colorectal cancer^19^. Whether LT has a negative impact on quality of life (QoL) has not been determined.

Liver resection and treatment of peritoneal metastases with cytoreductive surgery and hyperthermic intraperitoneal chemotherapy result in reduced QoL lasting 3–6 months after treatment^20^ ^21^. Before LT is offered to selected patients with non‐resectable colorectal cancer it is important to document that LT does not result in long‐term reduction in QoL. Short‐term QoL results for ten patients followed for up to 12 months after LT have been described^22^. The present study sought to describe long‐term QoL after LT in patients with non‐resectable CRLM without extrahepatic disease, based on assessments at inclusion and up to 3 years after LT of all 23 patients included in the LT trial (SECA‐I study).

## Methods

The SECA‐I study was an open prospective pilot study of LT in patients with non‐resectable liver‐only metastases from colorectal cancer. The study obtained approval from the Regional Ethics Committee and Institutional Review Board, and was registered in ClinicalTrials.gov (NCT01311453) before inclusion of patients. The primary endpoint of the study was OS at 2 years after LT; secondary endpoints included disease‐free survival and QoL evaluation. The first patient was transplanted in November 2006 and the last included patient in April 2012. The inclusion criteria have been described previously^23^. The main inclusion criteria were patients with non‐resectable CRLM without extrahepatic disease and good performance status (Eastern Cooperative Oncology Group grade 0–1). The immunosuppressive treatment used in the study comprised induction with basiliximab (interleukin 2 receptor antibody) and thereafter patients were maintained on an immunosuppressive regimen containing sirolimus (mTOR inhibitor), mycophenolate mofetil (inosine monophosphate dehydrogenase inhibitor) and corticosteroids. Corticosteroid treatment was tapered to zero in the course of the first 6 months after surgery.

QoL was assessed at baseline, and 3, 6, 12, 18, 24, 30 and 36 months after LT, using the European Organisation for Research and Treatment of Cancer (EORTC) QLQ‐C30 questionnaire version 3.0. The results obtained at the different time points were compared with baseline values. EORTC QLQ‐C30 is a self‐administered and multidimensional questionnaire that contains 30 items covering health issues relevant to patients with cancer; it includes a two‐item global health status scale (GHS), five function scales (physical, cognitive, emotional, social and role), three symptom scales (fatigue, pain and nausea/vomiting) and six single items (dyspnoea, sleep disturbance, appetite loss, constipation, diarrhoea and financial impact). The responses were transformed linearly to range from 0 to 100, and related items were transformed to function or symptom scales according to the manual^24^. A high function score indicates good function, whereas a high symptom score indicates more symptoms. A change in 10 points or more on the 0–100 scale was considered clinically significant^24^. The EORTC QLQ‐C30 has been translated into several languages and tested for psychometric properties in a number of countries, including Norway^24^. Questionnaires were completed by the patients before surgery (baseline) and were responded to by mail at the time points after transplantation. Patients who did not return questionnaires were reminded by a telephone call.

### Statistical analysis

Data were registered continuously in case report forms. QoL data are presented as mean values, and differences over time were evaluated by non‐parametric related‐samples Wilcoxon signed‐rank test. Between‐group differences were determined by independent‐samples *t* test. Survival data were estimated using the Kaplan–Meier method, and outcomes between groups compared using log rank tests. For all tests, two‐sided *P* < 0·050 was considered statistically significant. Analyses were performed using SPSS® version 21 (IBM, Armonk, New York, USA).

## Results

A total of 23 patients received LT according to the study protocol in the SECA‐I study. Patient characteristics are shown *Table* 1. In general, at inclusion the patients had good GHS and function scores with low symptom scores, although they had considerable liver metastases (*Table* 1).

**Table 1:** Baseline characteristics of patients in SECA‐1 trial

	No. of patients* (*n* = 23)
Age (years)†	54·7 (44·5–64·7)
Sex ratio (F : M)	10 : 13
Tumour site	
Colon	13
Rectum	10
Timing of metastasis	
Synchronous	19
Metachronous	4
Liver resection before LT	4
Chemotherapy before LT (no. of lines)	
1	10
2	9
3	4
> 10 liver metastases	8
Largest lesion > 5 cm	10
CEA > 5 μg/l	14

* Unless indicated otherwise.

† Values are median (range). LT, liver transplantation; CEA, carcinoembryonic antigen.

### Quality of life

The numbers of patients responding to the questionnaire of patients alive at baseline, 3, 6, 12, 18, 24, 30 and 36 months were 23 of 23, 22 of 23, 21 of 23, 22 of 22, 21 of 21, 19 of 21, 15 of 18 and 16 of 16 respectively.

**Figure 1:**
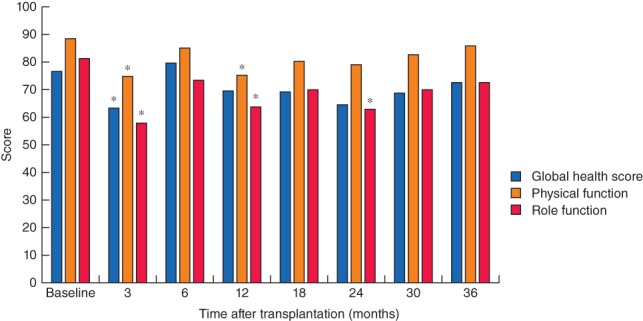
Mean global health, physical function and role function scores at baseline and up to 3 years after liver transplantation. Number of patients responding to the quality‐of‐life questionnaire at each time point: baseline, 23 of 23; 3 months, 22 of 23; 6 months, 21 of 23; 12 months, 22 of 22; 18 months, 21 of 21; 24 months, 19 of 21; 30 months, 15 of 18; and 36 months, 16 of 16. *P < 0·050 *versus* baseline (Wilcoxon signed‐rank test)

**Figure 2:**
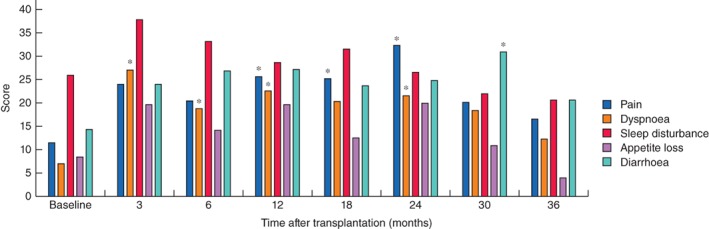
Symptom scores for pain, dyspnoea, sleep disturbance, appetite loss and diarrhoea at baseline and up to 3 years after liver transplantation. Number of patients responding to the quality‐of‐life questionnaire at each time point: baseline, 23 of 23; 3 months, 22 of 23; 6 months, 21 of 23; 12 months, 22 of 22; 18 months, 21 of 21; 24 months, 19 of 21; 30 months, 15 of 18; and 36 months, 16 of 16. *P < 0·050 *versus* baseline (Wilcoxon signed‐rank test)

At 3 months after LT, the patients had a significant and at least 10‐point decrease in mean GHS, physical function and role function scores (*Fig*. 1). Of the 23 patients, ten and 12 had decreases of 10 points or more in GHS and physical function scores respectively. Among the ten patients with such decreases in GHS score, four had Clavien–Dindo complication grades III–IV, whereas seven of 12 patients with decreased physical function scores had grades III–IV. Patients also reported significant and clinically relevant worsening in the symptom score for dyspnoea (*Fig*. 2).

GHS, physical function and role function scores were comparable to baseline scores (difference less than ± 10 points) at 6 months after LT (*Fig*. 1). Although GHS and role function scores were reduced by more than 10 points at some times during follow‐up, by 3 years the reported scores on the GHS and different function scales were similar to baseline values (*Fig*. 1). Cognitive, emotional and social function scores were also similar to baseline values at all time points after LT (data not shown).

At 6 months there was no clinically relevant and statistical difference compared with baseline in sleep disturbance, appetite loss, pain and diarrhoea, but the symptom score for dyspnoea was still worse than baseline values (*Fig*. 2). The mean values for pain, dyspnoea and diarrhoea were increased by more than 10 points at some time points from 12 to 30 months after LT. At 3 years after transplantation, these symptom scores were similar to baseline values. Symptom scores for fatigue, nausea/vomiting, constipation and financial impact were no different from baseline scores at any time point following LT (data not shown).

### Quality of life and survival

**Figure 3:**
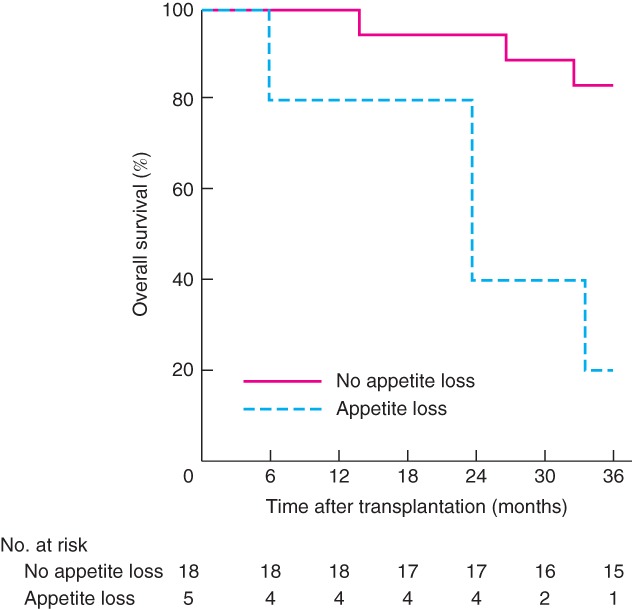
Kaplan–Meier estimated overall survival up to 3 years after liver transplantation related to symptom scores for appetite loss at baseline. *P* = 0·002 (log rank test)

**Figure 4:**
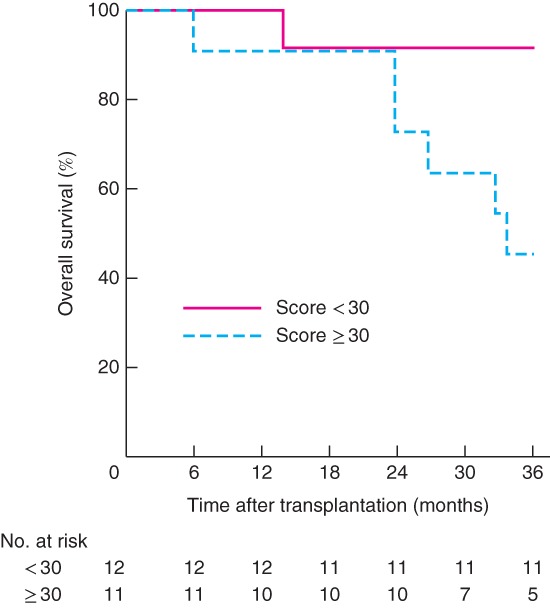
Kaplan–Meier estimated overall survival up to 3 years after liver transplantation related to fatigue symptom scores at baseline. Patients were divided into two groups with fatigue score of less than 30, and 30 or above. *P* = 0·023 (log rank test)

Seven patients died within 3 years of LT. Symptom scores for fatigue, pain and appetite loss at baseline were all significantly related to OS at 3 years after LT. Patients who died within 3 years after transplantation had significantly higher scores for all these symptoms at baseline. For patients who had died or were alive 3 years after LT, baseline scores were 39·7 and 21·5 respectively for fatigue (*P* = 0·033), 23·8 and 6·3 for pain (*P* = 0·032), and 23·8 and 2·1 for appetite loss (*P* = 0·005). OS was significantly reduced in patients with appetite loss (*P* = 0·002) (*Fig*. 3) and patients with a fatigue score of 30 or more (*P* = 0·023) (*Fig*. 4) at baseline.

Five patients had appetite loss and also had a pain score above 17 (mean 30) and fatigue score greater than 33 (mean 54). The patients with appetite loss at inclusion all had progressive disease on chemotherapy at the time of LT. In these five patients, the median size of the largest liver lesion was 90 mm and median carcinoembryonic antigen (CEA) level was 104 μg/l, compared with 46 mm and 8·5 μg/l respectively among patients who did not report appetite loss at inclusion.

Patients who died within 3 years after LT had a GHS score at inclusion in the study of 67·9 compared with 81·3 in patients who were alive at 3 years after LT, although this difference was not significant (*P* = 0·273). None of the function scores were significantly related to OS at 3 years and no items in the QoL questionnaire were significantly related to disease‐free survival at 12 months.

## Discussion

At 3 months after LT, patients had significantly worse GHS and physical function scores, along with worse symptom score for dyspnoea. These changes were all considered to be of clinical relevance. Function and symptom scores except those for dyspnoea had returned to baseline values 6 months after LT suggesting relatively rapid recovery from LT‐related problems. Decrease in health‐related QoL and physical function with full recovery within 6 months has also been reported after resection of CRLM^20^ ^25^. Short‐term reduced physical function and increased symptom scores have also been reported in patients with colorectal cancer treated by pelvic radiation therapy and abdominal surgery combined with intraperitoneal chemotherapy^21^ ^26^.

There was no significant and clinically important change from baseline in any function or symptom scale 3 years after LT, suggesting that the patients maintained good QoL for a long period of time and that the immunosuppressive treatment used in this study did not have a negative impact on reported QoL. In contrast, patients undergoing LT for chronic liver failure have reported reduced GHS and physical function scores compared with the general population. It has been suggested that this may be due to immunosuppressive therapies^27^.

Although the reported symptom scores in the whole cohort were low, patients who died within 3 years of transplant reported significantly higher scores for fatigue, pain and appetite loss at baseline. Those with any appetite loss or a fatigue score of at least 30 had significantly lower 3‐year survival rates than those without appetite loss or with fatigue score below 30. These observations may suggest that patients with general symptoms related to the malignant disease have reduced OS after LT. It has been shown previously that patients with larger colorectal tumours (exceeding 5·5 cm) or CEA levels over 80 μg/l at time of LT have reduced OS^19^. In the present study, patients with general symptoms also had a bigger largest lesion and higher CEA levels suggesting more advanced disease at the time of LT. This suggests that symptom score may be incorporated into the selection process for LT in patients with colorectal cancer. QoL scoring has also been shown to be related to OS in patients with head and neck cancer receiving curative radiation therapy^28^, and asymptomatic patients with colorectal cancer have increased OS after starting palliative chemotherapy compared with patients reporting various disease‐related symptoms^29^.

QoL evaluation in this study was performed using the generic cancer instrument EORTC QLQ‐C30 covering GHS, physical function, social function, cognitive function and role function as well as symptom scores for pain, dyspnoea, sleep disturbance, appetite loss and diarrhoea. The EORTC QLQ‐CR38 colorectal questionnaire covers function scales including body image and sexuality, and symptom scales for micturition problems, gastrointestinal problems, chemotherapy‐related side‐effects, defaecation problems, stoma‐related problems and sexual problems. These functions and symptoms are not covered by the EORTC QLQ‐C30 questionnaire and mainly relate to the primary surgery rather than transplantation. Nevertheless, they may still be relevant issues in these patients.
